# Thoracoscopic lobectomy with mediastinal lymph node dissection as a standard surgery for T1-2N0M0 non-small cell lung cancer (>300 surgeries experience)

**DOI:** 10.1016/j.amsu.2018.09.022

**Published:** 2018-09-26

**Authors:** Arif Allakhverdiev, Mikhail Davydov, Parvin Akhmedov

**Affiliations:** Department of Thoracic Surgery, N.N. Blokhin Cancer Research Center, Kashirskoe Road 23, 115478, Moscow, Russian Federation

**Keywords:** Non-small cell lung cancer, Thoracoscopic lobectomy, VATS, Mediastinal lymph node dissection

## Abstract

**Background:**

A lot of clinics worldwide in recent years recommend the use of minimally invasive surgical procedures in the early stages of lung cancer claiming that this technique helps reduce the number of postoperative complications, shortens the period of social rehabilitation of patients, without significantly affecting the long-term results of treatment. In this study we evaluate immediate and long-term results of surgical treatment of patients with early stages of non-small cell lung cancer (NSCLC) after video-assisted thoracoscopic lobectomy (VATS) with mediastinal lymph node dissection.

**Materials and methods:**

Since 2008 317 patients with T1-2N0M0 NSCLC over 20 (median age was 65.3 ± 2.5) years underwent VATS with mediastinal lymphadenectomy. Total number of men was 186 (58.7%), women – 131 (41.3%). Histologically verified adenocarcinoma was in 278 (87, 7%), Squamous cell carcinoma in 39 (12.3%). A group of patients who underwent thoracotomy lobectomy (n = 189) was taken to compare immediate and long-term results. Median age in this group was 66.5 ± 1.7. Total number of men was 115, women – 74. Histologically verified adenocarcinoma was in 154 (82.4%), Squamous cell carcinoma in 35 (17.6%).

**Results:**

Conversion to thoracotomy during VATS was in 14.3% of surgeries. There was no postoperative mortality in VATS group, whereas in open surgeries this happened in 2.6%. The 3 and 5-year overall survival (OS) rate was 94.0% and 94.0% in the VATS group respectively, 83.0% and 78.0% in the thoracotomy group for clinical stage T1N0M0 NSCLC (p = 0.04554).

**Conclusion:**

Considering the results of our research and the literature review we made sure that VATS lobectomy with mediastinal lymph node dissection is an alternative procedure to open approaches: it is much safer, reduce the frequency of post-operative complications and the rehabilitation period. We believe that complete VATS lobectomy with mediastinal lymph node dissection must be taken as a standard in surgical treatment of patients with early stages of non-small cell lung cancer.

## Highlights

• Thoracoscopic Lobectomy reduces the frequency of postoperative complications.
• Mediastinal Lymph Node Dissection is a standard procedure in early stages of non-small cell lung cancer.
• Locoregional recurrence is much higher in group of patients underwent open surgery comparing with thoracoscopic surgery.

## 1 Introduction

Thoracoscopic Lobectomy (TL) was first mentioned in the literature at the beginning of the 1990s [6]. Over time TL was widely used in the surgical treatment of Non-Small Cell Lung Cancer (NSCLC) and many studies have been published [2,10,21]. However, the safety of performing TL and long-term oncological results still cause concern for most surgeons. This fact explains why according to the Association of Thoracic Surgeons TL is performed in only 30% of patients undergoing lobectomy for lung cancer [1]. There are no large randomized studies and many published studies in the literature are mainly conducted on a heterogeneous group of patients including oncological, specific and inflammatory lung diseases. In a number of studies incorrect analyses were performed: results of surgical treatment were compared with results of patients who received combined therapy. Our work is devoted to a comparative analysis of immediate and long-term results of treatment of patients with early stages of NSCLC after TL and open surgery lobectomy (OSL).

## 2 Materials and methods

**Fig. 1:**
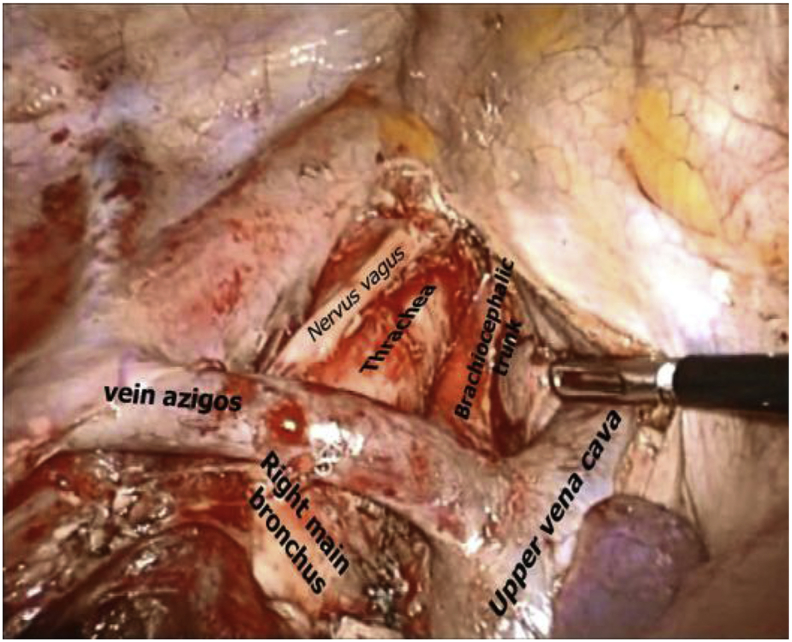
Thoracoscopic paratracheal lymphadenectomy.

**Fig. 2:**
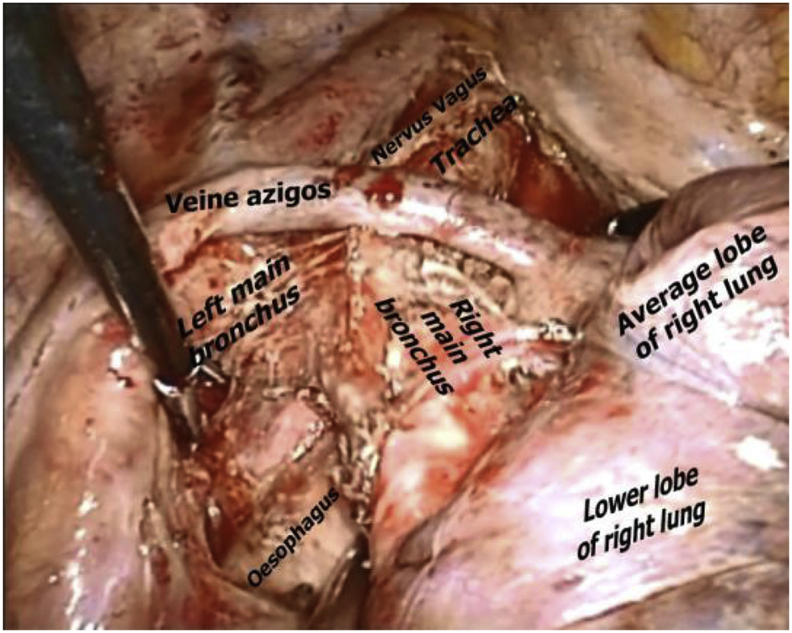
Thoracoscopic Lymph node dissection of the bifurcation.

Since 2008 317 patients with T1-2N0M0 NSCLC underwent TL with mediastinal lymph node dissection in thoracic department of XXXXXX Cancer Research Center. All surgeries were accompanied by lymphodissection of the hilum of the lung and mediastinum. Regardless of the location of the primary tumor lymphodissection of the upper and lower mediastinum was performed. The area in thoracic cavity after thoracoscopic mediastinal lymph node dissection is shown in Fig. 1, Fig. 2.

A group of 189 patients with a similar stage of NSCLC underwent OSL was used to compare long-term results. The characteristics of the compared groups are given in Table 1.

**Table 1:** Distribution of parents depending on the type of surgical approach.

Type of surgical approach	Number of patients	Age	Gender		Adenocarcinoma	Squamous cell carcinoma
			Male	Female		
Thoracoscopy	317	65.3 ± 2.5	186	131	278(87.7%)	39(12.3%)
Thoracotomy	189	66.5 ± 1.7	115	74	154(82.4%)	35(17.6%)

There were no statistically significant differences in the compared groups. We use a program Statistica 6.0 to analyze immediate and long-term results.

## 3 Results

Conversion to thoracotomy was in 14.3% of cases in the group of patients underwent TL. Postoperative mortality rate after thoracoscopic approach was absent, whereas, after OSL it was 2.6%.

In 6 (2.8%) patients after TL prolonged bleeding through pleural drainage was noted. Two of them were taken in an emergency surgical room to make a re-thoracoscopy, suturing the pulmonary parenchyma and 4 patients underwent chemical pleurodesis. A similar complication in the group of patients operated from thoracotomy approach was observed in 11 (5.8%) patients. The average duration of standing of pleural drainages in the TL group was 3.8 days, versus 5.7 days after OSL. The average length of stay in the hospital after thoracoscopic approach was 7.3 days, whereas after thoracotomy approach this period was 13.3 days. 3 and 5-year overall survival (OS) in the general group of patients with.

**Fig. 3:**
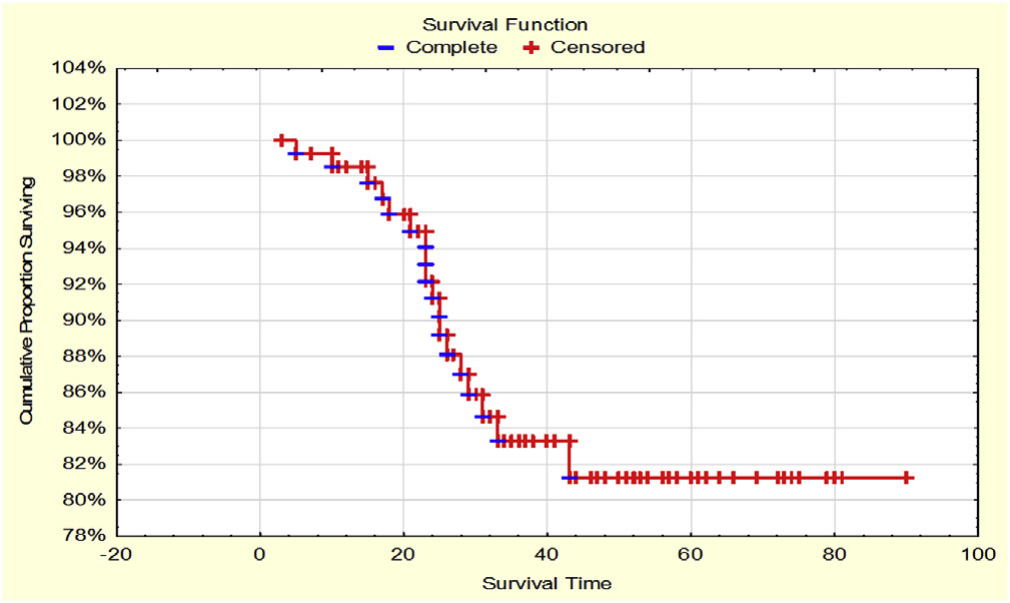
OS of patients with T1-2N0M0 NSCLC.

T1-2N0M0 NSCLC was 86% and 81% respectively (Fig. 3).

**Fig. 4:**
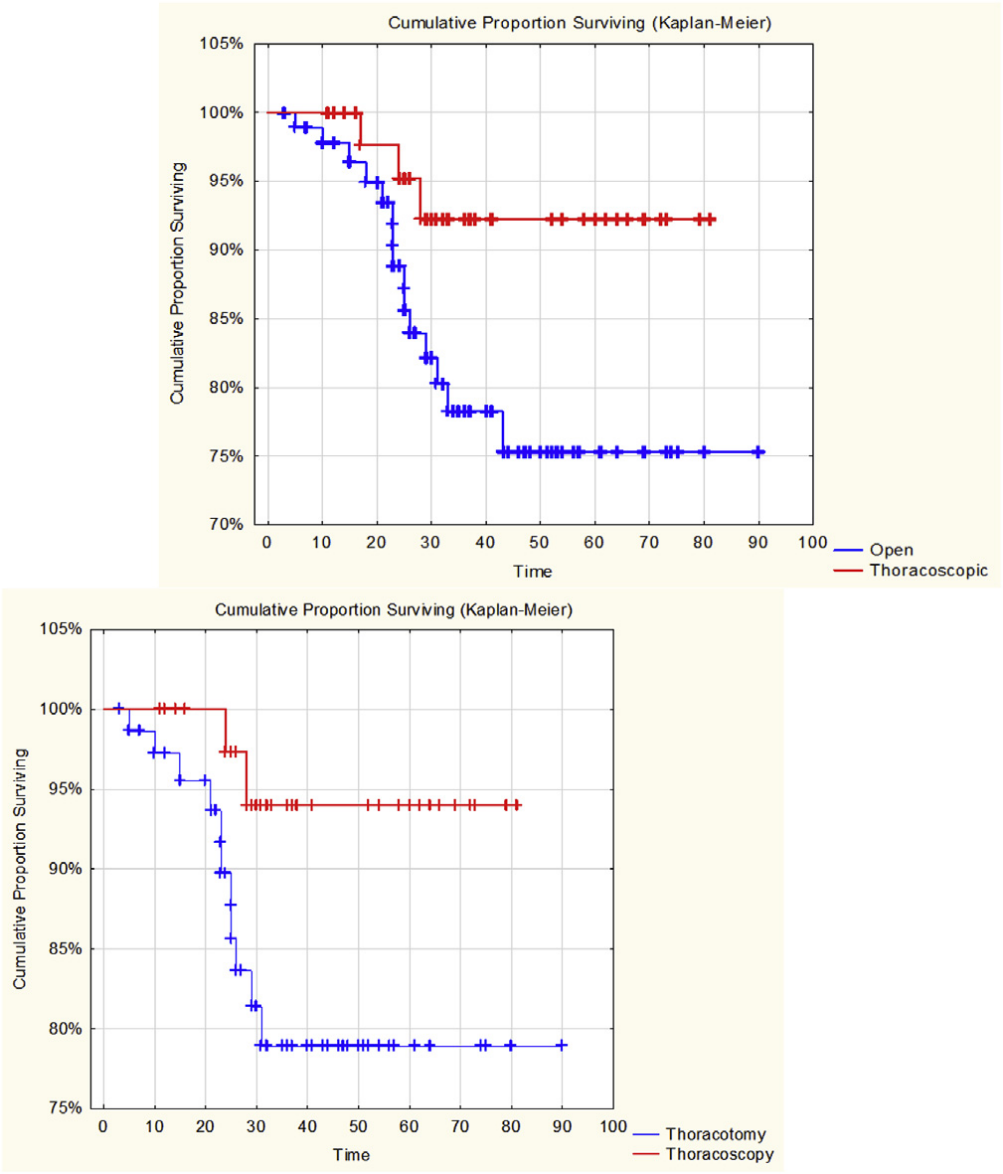

We compare long-term results after TL and OLS depending on the type of surgical approach in patients with T1-2N0M0 NSCLC. The results are as follows: 3 and 5-year overall survival after TL was 93% and 93% respectively, after OLS the results were 82% and 75% respectively. (Test statistic = 1,955203, p = , 05056, Log-Rank Test), Fig. 4.

**Fig. 5:**
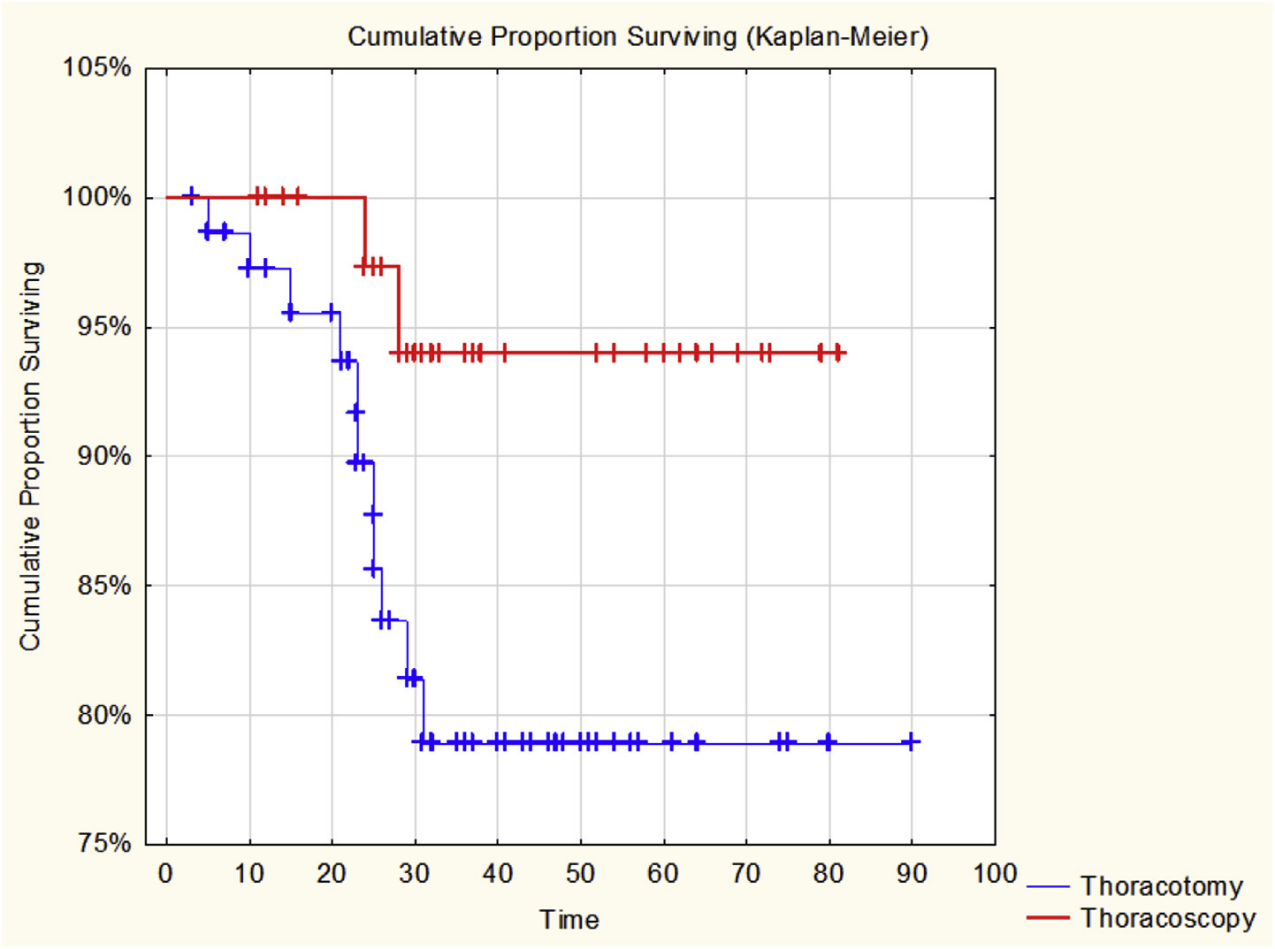

Compare the long-term results in the group of patients with prevalence of T1N0M0 the following results were obtained: 3 and 5-year survival after TL was 93% and 94%, after OSL 83% and 78% respectively. (Test statistic = 1.999667, p = , 04554, Log-Rank Test), Fig. 5.

## 4 Discussion

Our study demonstrates the advantages of TL in comparison with OSL, which were expressed by a decrease in the number of postoperative complications, a decrease in the duration of standing of pleural drainages and a reduction of the hospital stay. There was no statistically significant difference in the duration of the surgery. Whitson et al. reported the results, which did not contradict our data [20]. 39 studies were included in the review, 3256 patients underwent TL and for 3114 patients were performed thoracotomy approach. 10 out of 39 studies were devoted to comparative analyses of both methods of surgery. In these studies, thoracoscopic approach were characterized by shorter postoperative standing of pleural drainages (4.2 days compared with 5.7 days, P = 0.025) and shorter hospital stay (8.3 days compared with 13.3 days, P = 0.016) compared with patients underwent OSL.

Another systematic literature review comparing TL and OSL showed vague advantages of group of patients underwent thoracoscopic approach. However, the data obtained in this meta-analysis included non-uniform groups of compared patients and the resulting outcomes were not correct [22]. Analyses of some studies showed no significant differences in intraoperative blood loss, pleural drainage standing and postoperative hospital stay depending on the performed surgery. However, there were statistically significant differences in the duration of surgical procedure, which was less in the group of patients, underwent thoracotomy lobectomy [12,14,15,23]. In other studies significant differences in the duration of surgery depending on the type of approach were not revealed and the results were comparable [7,11,17,18]. The main factor affecting the duration of thoracoscopic surgery is the qualification of the surgeon. There is a learning curve for thoracoscopic surgeries and the resulting contradictory literature data, in our opinion, is primarily related to differences in the qualifications of surgeons.

Analyses of the literature shows that postoperative complications in patients who underwent TL in comparison with patients underwent OSL were significantly lower [22]. We also studied the frequency of postoperative complications after thoracoscopic and open approaches surgeries. Significant differences were obtained in the frequency of postoperative bleeding, the development of atrial fibrillation and pneumonia, which were observed more often in patients after thoracotomy. The explanation for this fact we find in the following: firstly, thoracoscopic approach is much less traumatic, unlike open approach. Some studies have demonstrated that patients underwent thoracoscopy lobectomy had a much less postoperative pain syndrome and better preserved pulmonary function, which facilitated the fastest recovery and reduced postoperative therapeutic complications [3,5,12]. Secondly, minimally invasive surgeries in comparison with open surgeries have less effect on immunosuppression. According to the literature a decrease in the inflammatory response of the body was revealed after thoracoscopic surgeries compared with open surgeries [13,23]. Particularly, thoracoscopic surgeries are associated with a decrease in the release of inflammatory (IL-6, IL-8) and anti-inflammatory (IL-10) cytokines in comparison with surgeries performed from thoracotomy approach [8]. This fact can cause a decline in the development of pneumonia in the postoperative period.

The main criterion for assessing the effectiveness of treatment of cancer patients is OS. In our study the 5-year overall survival rate of patients with T1-2N0M0 NSCLC after TL was - 94%. According to the literature, long-term results after TL for lung cancer accounted for 75–94.9%. Yan et al. [22], reported a significant improvement in 5-year survival in the group of patients after TL compared with OSL (95% CI, 0.45–0.97, P = 0.04). Another systematic meta-analysis also showed an improvement in overall 5-year survival after thoracoscopic surgeries for the early stage of NSCLC (p = 0.003). Reliability of the differences in results appeared from the 4-year follow-up. However, there were no statistically significant differences in 1 and 3-year overall survival [20].

The frequency of locoregional recurrences is also a criterion for evaluating the long-term results of treatment of cancer patients. A meta-analysis of the frequency of locoregional recurrences in the thoracoscopic and open lobectomy groups for the first stage of NSCLC did not reveal statistically significant differences [9]. The results of this study don't contradict the analysis reported by Yan et al. [22]. The author revealed a lower frequency of locoregional reoccurrences in the group of patients after TL in comparison with OSL, although no statistical reliability was obtained.

We have a question: why after TL the long-term results are higher and the frequency of locoregional reoccurrences does not exceed those in comparison with OSL? It is impossible to give a simple answer to this question. In our opinion, the main source of the results is the quality of the lymph node dissection performed. Some studies have demonstrated that the quality of performing mediastinal lymph dissection in TL is not inferior to that of OSL [16,19]. In addition, less trauma leads to a faster recovery of patients and allows earlier begging of adjuvant therapy, which can improve long-term outcomes. Jiang et all. [4], demonstrated that patients who underwent by thoracoscopic surgeries recovered more quickly in the postoperative period, have better compliance and fewer delayed or reduced dose on adjuvant chemotherapy than those patients underwent by thoracotomy surgeries. It can be stated that thoracoscopic approach in comparison with thoracotomy approach in early stages of NSCLC reduce the frequency of postoperative complications, reduce immunosuppression, promote early beginning of adjuvant therapy, improve social rehabilitation of patients and improve long-term results of treatment. The last fact is difficult to explain and in our opinion further studies are required for its interpretation.

## 5 Conclusion

Considering the results of our research and the literature review we made sure that VATS lobectomy with mediastinal lymph node dissection is an alternative procedure to open surgeries: it is much safer, reduce the frequency of post-operative complications and the rehabilitation period. We believe that complete VATS lobectomy with mediastinal lymph node dissection must be taken as a standard in surgical treatment of patients with early stages of NSCLC.

## Provenance and peer review

Not commissioned, peer reviewed.

## Conflicts of interest

Arif Allakverdiev has no conflict of interests.

Mikhail Davydov has no conflict of interests.

Parvin Akhmedov has no conflict of interests.

## Sources of funding

No sources of funding was provided.

## Ethical approval

Study was exempt from ethical approval from Russian Cancer Research Center N.N. Blokhin.

## Unique identifying number

Researchregistry4188.

## Author contribution

Arif Allakverdiev has made the design of this study, collected the database, analyzed and wrote this article.

Mikhail Davydov has made the design of this study, collected the database, analyzed and wrote this article.

Parvin Akhmedov has analyzed and wrote this article.

## Guarantor

Arif Allakverdiev is guarantor, who accepts full responsibility for the work, has access to the data, and controlled the decision to publish.
